# Bioinspired Pressure Sensitive Adhesives Based on Natural Deep Eutectic Solvents

**DOI:** 10.1021/acs.macromol.5c01360

**Published:** 2025-08-18

**Authors:** Abinaya Arunachalam, Elise Le Cornec, Julien Es Sayed, Marleen Kamperman

**Affiliations:** † Zernike Institute for Advanced Materials, 3647 University of Groningen, Nijenborgh 3, 9747 AG Groningen, The Netherlands; ‡ Biofabrication and Bio-Instructive Materials, Biotechnology Center, The Silesian University of Technology, B. Krzywoustego 8, Gliwice 44-100, Poland

## Abstract

Petroleum-based pressure sensitive adhesives (PSAs) have been ruling the industry with their rigorous standards for adhesion strength and durability. While petrochemical PSAs have been the center of attention for many years, there is growing interest in developing biobased alternatives. In this study, a bioinspired PSA was developed combining natural deep eutectic solvents (NaDES) and hyaluronic acid, a biosourced polysaccharide, which has not been previously explored in PSA formulations. By leveraging the unique properties of NaDES, these soft materials derive their adhesive properties primarily from hydrogen bonds and physical cross-links arising from polymer chain entanglements. We investigate the role of hyaluronic acid in creating an elastic network within the viscous hydrogen-bonded NaDES, demonstrating tunable mechanical properties that depend on molecular weight and concentration. In parallel, we predict the adhesive properties of these materials from small amplitude oscillatory shear measurements. While such predictions show strong correlation with our experimental results, it remains necessary to support these predictions with nonlinear tack measurements, as such frameworks may not fully capture the behavior of soft supramolecular adhesives. Overall, the bioinspired adhesives presented in this work demonstrate tunable adhesion properties, highlighting their potential for further development as sustainable PSAs.

## Graphical abstract

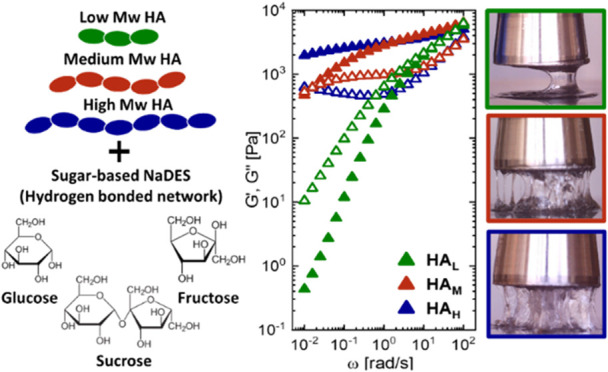

## Introduction

Pressure sensitive adhesives (PSAs) are a class of adhesives that are of specific interest due to their ability to adhere to a substrate under the application of low pressure.^1^ Such adhesives are obtained through an optimized balance of liquid-like and solid-like properties, which are crucial for their performance. These materials possess sufficient liquid-like properties to create optimal contact with the substrate and facilitate dissipation of energy during debonding.^2,3^ On the other hand, the highly entangled and cross-linked structure contribute to solid-like properties, thus enabling them to have high shear and creep resistance.^4,5^ While synthetic PSAs have been the industry standard for many years, sustainability concerns are becoming increasingly important.^6–8^

In this context, biobased PSAs have been widely explored.^9^ One popular strategy to prepare biobased acrylic PSAs is the use of acrylic and methacrylic acid derived from renewable sources.^6^ Other bioresources used for this purpose include natural rubber, cellulose, lignin, and vegetable oils.^7,10,11^ Biobased and bioinspired PSAs offer the potential to reduce the reliance on petroleum-based materials, and are especially interesting for biomedical applications, due to the use of biopolymers that can improve biocompatibility and bioactivity.^9,12^ In this regard, natural deep eutectic solvents (NaDESs) are an emerging class of green solvents that have untapped potential in the field of biobased and bioinspired PSAs. Our system is inspired by the exudates of carnivorous plants, particularly *Drosera* species, which secrete a sugar-rich PSA to capture and immobilize insects.^13^ This bioinspired approach was previously introduced in our earlier work as an environmentally friendly pest control technique.^14^ The exudates of *Drosera* have been reported to manifest as a NaDES containing approximately 4 wt % of an acidic polysaccharide with a molecular weight exceeding 2 MDa, composed of a mixture of sugars such as arabinose, xylose, mannose, galactose, and glucuronic acid, along with *myo*-inositol, inorganic cations including Ca^2+^, K^+^, and Na^+^, and a high water content of around 95%.^15–22^

NaDESs are a class of deep eutectic solvents (DESs) formed as a result of hydrogen bonds between a hydrogen bond donor and an acceptor at a specific molar ratio, termed the eutectic composition.^23^ NaDES and DES differ primarily in the constituents where the former is composed of natural substances, mainly primary metabolites like amino acids and sugars.^24,25^ Sugar-based NaDESs exploit the hydrogen bonding ability of sugars resulting in a material with high viscosity which is often seen as a disadvantage for several applications.^26^ However, we use the high viscosity of sugar-based NaDES to our advantage to develop viscoelastic PSAs.

Historically, concentrated sugar solutions have been used as adhesives for various purposes with early uses ranging from simple household repairs to more specialized industries such as in woodworking.^27^ This is attributed to their enhanced ability to form hydrogen bonds with water leading to an increase in viscosity and consequently, stronger cohesive forces.^28^ Several sugar-based materials such as glucose, sucrose, starch, and sugar alcohols have been researched to prepare different kinds of adhesives.^29–31^ However, a key limitation of these materials is that they generally require a drying step to form effective bonds, as they do not adhere well through continuous contact in their liquid state. The simplicity and effectiveness of sugar-based adhesives along with their ease of scalability have made them an attractive option for the development of biobased and sustainable adhesives. Sugars and organic acids have also been researched under the context of forming wood adhesives.^32–35^ Li et al. reported one such adhesive made from citric acid and glucose prepared at a high temperature of 110 °C where the adhesive properties were attributed to both covalent and noncovalent interactions.^36^ The main factor contributing to adhesiveness was considered to be covalent cross-links arising from the esterification reaction between citric acid and glucose. Wu et al. studied adhesives also prepared from sugars and acids at lower temperatures.^35^ They experimentally proved the absence of any covalent interactions in the adhesive. The adhesiveness of this material was attributed solely to hydrogen bonds between the sugar, acids and water forming a viscous deep eutectic solvent.^35^ While not traditionally studied as PSAs, the properties of these sugar-based DES systems suggest that they could potentially be adapted for PSA applications.

This work presents a bioinspired PSA that functions without the need for a drying step, unlike typical sugar-based adhesives. Here, we characterize the viscoelastic behavior of PSAs made with hyaluronic acid (HA) dissolved in a sugar-based NaDES. HA in this context, serves the purpose of forming an entangled network within the solvent. By incorporating HA into the sugar-based NaDES, we aim to understand how its concentration and molecular weight and subsequently, the polymer network influences the adhesive’s performance, particularly in terms of viscosity, tack, and cohesion. While synthetic PSAs are largely dependent on chemical cross-linking, our work focuses on a natural system where the adhesive properties are influenced by physical cross-links arising from high concentrations of HA rather than chemical cross-links. We attempt to establish the link between linear rheology and tack using predictive concepts that have been traditionally established for acrylic PSAs. By evaluating the accuracy and applicability of these techniques, in the context of our sugar-based PSAs, we seek to contribute valuable insights that could aid the development and optimization of sustainable, environmentally friendly adhesives.

## Materials and Methods

### Materials

Hyaluronic acid sodium salt (HA) with average molecular weights of 30–50 kDa, 200–400 kDa, and 750–1000 kDa (denoted as HA_L_, HA_M_, and HA_H_ respectively) were purchased from Glentham Life Sciences Ltd. (Corsham, U.K.). For the preparation of the NaDES, d-glucose and sucrose were purchased from Sigma-Aldrich (Darmstadt, Germany) along with d-fructose from Boom B.V. and were used without any further purification. Deionized water, produced by reverse osmosis, was used for all experiments as required. All chemicals were used as received without further purification.

### Preparation of Bioadhesive

The NaDES was prepared by stirring glucose, fructose, sucrose, and water, in a fixed molar ratio of 1:1:1:11 at 65 °C for 1 h until a clear liquid was obtained. The NaDES is termed GFSH hereafter (corresponding to G-glucose, F-fructose, S-sucrose, and H-water). The solvent was used if no crystallization was observed after 1 day. HA was dispersed in 5 g of GFSH to attain the desired polymer concentration. This dispersion was homogenized through vortexing for a duration of 10 s, followed by centrifugation for 4 min at 3000 *g* to help better dispersion of HA in the NaDES. The mixture was then heated at 65 °C for 1 h to facilitate the dissolution of the polymer within the solvent matrix. The samples were centrifuged immediately at 3000 *g* for 5 min while they were still hot, to eliminate any trapped air bubbles. Subsequently, the samples were allowed to cool gradually to room temperature over a 24 h period before subsequent experimental analyses. All samples were used for further experiments within a maximum storage period of 1 week.

In order to prepare samples with HA concentration greater than 6 wt %, a heating protocol was employed to prepare homogeneous materials. The GFSH solvent was diluted with water to attain a ratio of 65:35 GFSH/water, in order to release some water from the solvent to facilitate further HA dissolution. HA was then added as described above at the required concentration followed by vortexing, centrifuging, and heating at 65 °C for 1 h until homogeneous. After dissolution, the samples were allowed to continue heating at 65 °C in an open vial for 24 h to allow evaporation of excess water. This technique enabled the dissolution of high concentrations of HA without practicality problems arising from their very high viscosity.

### Rheological Measurements

The rheological characteristics of the HA/GFSH samples were investigated using oscillatory measurements using the Anton Paar MCR302e rheometer. Measurements were performed using a cone and plate geometry (25 mm diameter, 1° cone angle) for liquid like samples. For stiffer samples, a plate and plate geometry (25 mm diameter) was used. To establish equilibrium after loading samples, measurements were started when the normal force dropped below 0.1 N at 20 °C. Silicone oil was used as a trap around the gas–liquid interface to prevent the material from drying during measurement. An amplitude sweep was first carried out at 10 rad/s to determine suitable strain values for rejuvenation and aging. Consequently, the sample was rejuvenated to erase the mechanical history through a dynamic time sweep (DtS) at 10 rad/s with a strain value of 550% for 30 s followed by aging to recover the mechanical properties at 0.1% for 100 s. Following this, an amplitude sweep at 100 rad/s was used to determine the linear viscoelastic regime. Frequency sweeps were then performed in this regime from 100–0.1 rad/s to construct the viscoelastic spectra of the materials.

### Adhesion Testing

Probe tack tests were also performed on the Anton Paar MCR302e rheometer using a flat stainless-steel probe with a sand-blasted surface and a diameter of 10 mm (PP10/S). The bottom plate was fixed at 20 °C following which samples were loaded on to the rheometer either with a pipet for liquid-like samples or a spatula for more solid-like materials. In all measurements, the probe was lowered such that the sample thickness was brought to a gap size of 150 μm (*h*_0_). The sample was allowed to establish sufficient contact with the probe for 1 min following which the probe was retracted at a speed of 100 μm/s. A Toolcraft USB microscope was used to record the side view and bottom view of the samples during retraction. Brightness and contrast of the bottom view video frames were uniformly increased across all time points to improve visibility of morphological features. The normal force was recorded as a function of displacement which was later converted into stress vs strain curves. Nominal stress (σ), characterized as the measured force (*F*) divided by the initial contact area (*A*_0_) was plotted against nominal strain (ε), determined as the displacement of the probe normalized by the initial sample thickness (*h*_0_). Work of adhesion (*W*_adh_) was subsequently calculated as the product of the initial sample thickness, *h*_0_, and the area under the stress vs strain curves, calculated as an integral (*W*_adh_ = *h*_0_∫_0_^∞^σdε). The average of quadruplicate measurements for each sample was used for further analysis.

## Results and Discussion

### Material Composition

Sugars are inherently sticky materials due to their ability to form hydrogen bonds in water resulting in high viscous forces.^37^ Based on our previous studies, we prepared a sugar-based NaDES using glucose, fructose, sucrose, and water in a molar ratio of 1:1:1:11 (GFSH).^14^ This eutectic mixture exhibited a high inherent viscosity, possibly due to a dense hydrogen-bonding network, making it a promising candidate for adhesive applications.^14^ Although other sugar solutions such as glucose, fructose, water (GFH) and fructose, water (FH) are also perceived to be sticky, the lower density of hydrogen bonds in these mixtures results in insufficient strength to sustain applied stress. The GFSH solvent used in this study behaves as a Newtonian fluid with high viscosity as shown in Figure 1A, but demonstrates poor adhesion, as shown by the low area under the corresponding stress vs. strain curve in Figure 1B. The resulting forces during retraction are very weak and are primarily a result of capillary forces as can be seen from the side view images in Figure 1C. To impart elasticity to this system, HA, a polysaccharide composed of several repeating units of *N*-acetylglucosamine and glucuronic acid, was incorporated. The long linear nature of HA enables it to form a network-like entangled structure in a good solvent, thus forming a viscoelastic liquid with shear-thinning behavior (Figure 1A). The work of adhesion of the sample with 6 wt % HA_H_ in water, although higher than pure GFSH, due to the increased elastic nature (Figure 1B,1D), is still not appreciable enough. In contrast, the same concentration of hyaluronic acid combined with the NaDES composed of GFSH imparts the material with a gel-like behavior as can be seen from the complex viscosity having a strong angular frequency dependence with the absence of a Newtonian plateau (Figure 1A).^38^ A subsequent surge in the work of adhesion and the strain at break can also be observed (Figure 1B). This highlights the importance of having a balance between the viscous component of GFSH and the elastic contribution from hyaluronic acid, resulting in an adhesive with a viscoelastic structure that enables both sufficient dissipation and resistance to the applied stress as shown in Figure 1E.

**Figure 1:**
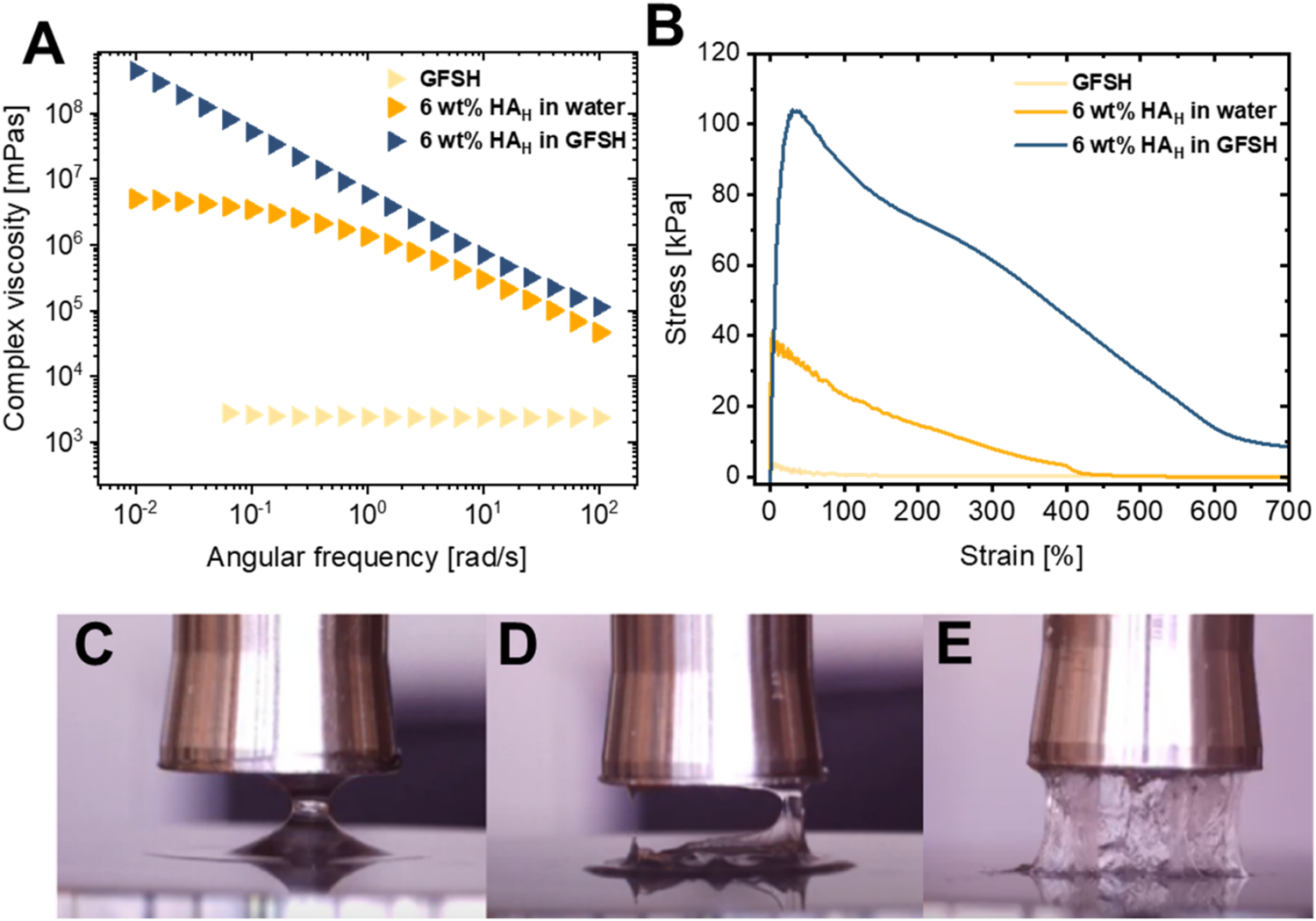
(A) Complex viscosity, (B) stress vs strain curves during retraction and their corresponding side view images for (C) GFSH solvent, (D) 6 wt % HA_H_ (750–1000 kDa) in water and (E) 6 wt % HA_H_ (750–1000 kDa) in GFSH.

### Viscoelastic Properties

In this section, we aim to explore the viscoelastic properties that HA imparts to a material when it is dissolved in a NaDES composed of GFSH. To gain deeper insights, we employ small amplitude oscillatory shear (SAOS) measurements, a well-established technique for examining the viscoelastic characteristics of complex solutions. As can be expected, the viscoelastic structure is highly dependent on the molecular weight and concentration of hyaluronic acid since that influences the network formation in the solvent. When HA with a low molecular weight of 30–50 kDa (HA_L_) is dissolved in GFSH, we observe that the loss modulus, *G*″, is above the storage modulus, *G*′, for the entire experimental frequency window except at concentrations of 4 wt % and above, where slight overlap of *G*″ and *G*′ is observed (Figure 2A). At all studied concentrations, *G*″ and *G*′ appear to have slopes close to 1 and 2 at low frequency, indicating a fully relaxed terminal regime. For HA concentration of 4 wt %, both *G*′ and *G*″ show a slope of 0.5 at high frequencies, dynamics expected for unentangled semidilute polymers in a good solvent predicted by the Rouse model.^39–41^ This indicates that the material’s mechanical response is dominated by viscous behavior, with minimal contribution from any elastic network formation as can be seen from the lack of entanglements. For concentrations below 4 wt %, we observe that the slopes of *G*′ and *G*″ at high frequencies are steeper than 0.5, reminiscent of dynamics predicted by the Zimm model, which describes the dynamics of polymer chains in dilute conditions where hydrodynamic interactions dominate.^42^ At the highest studied concentration of 6 wt %, we see the appearance of the characteristic crossover frequency, ω_d,_ at 6.31 rad/s = 1/τ_d_, where τ_d_ is the longest relaxation time of the system, the time in which the polymer chain has fully completed its reptation and overcome its entanglements.

**Figure 2:**
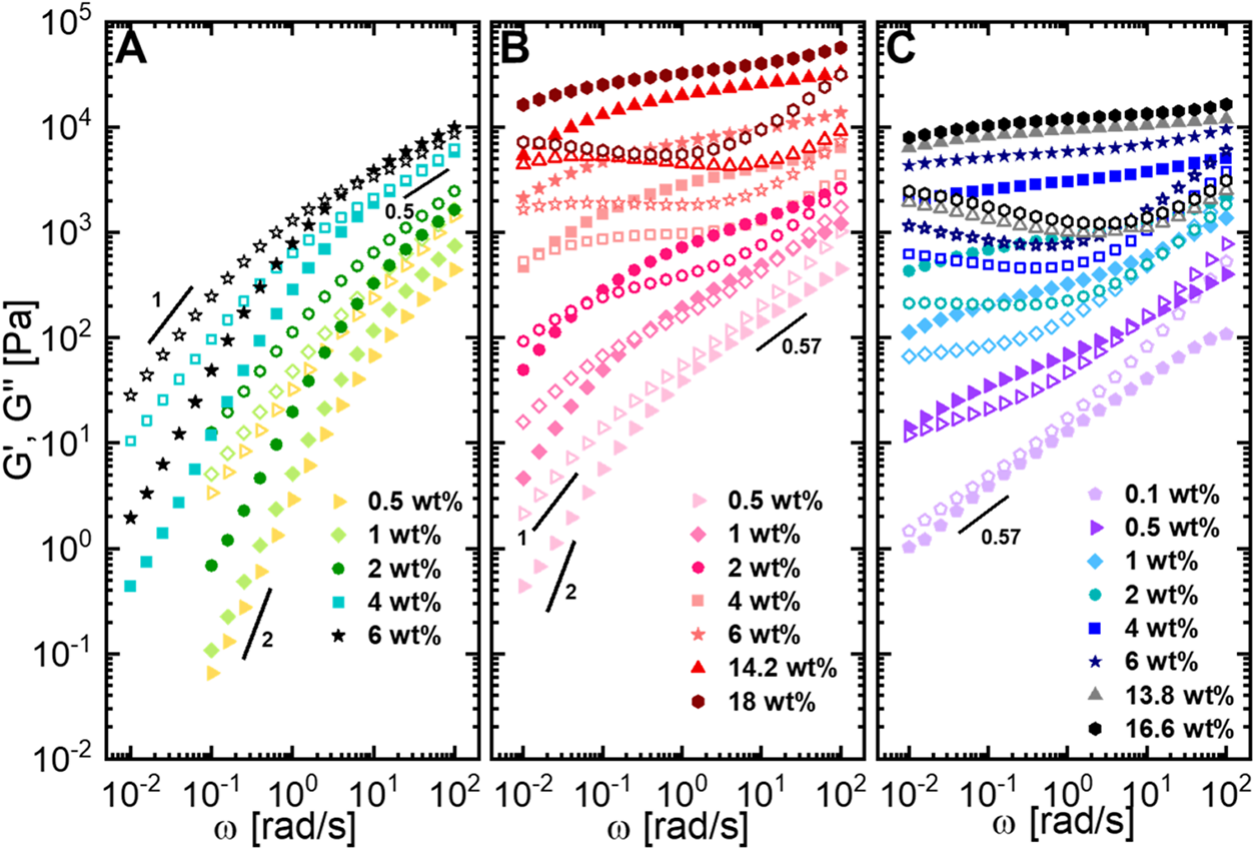
Linear rheology for varying concentrations of HA in GFSH with molecular weights of (A) 30–50 kDa (HA_L_), (B) 200–400 kDa (HA_M_) and (C) 750–1000 kDa (HA_H_).

Upon increasing the molecular weight of HA to a medium range of 200–400 kDa (HA_M_), we notice the appearance of a clear entanglement plateau from 1 wt % and above (Figure 2B). For the lowest concentration of 0.5 wt %, we also observe *G*″ being above *G*′ for the entire experimental window. Similar to the lower concentration range of HA_L_, we observe Zimm modes at high frequencies followed by a fully relaxed terminal flow. An increase in the concentration of HA_M_ displays an expected and corresponding increase in moduli accompanied by a broader entanglement plateau and a higher characteristic crossover frequency, ω_e_ = 1/τ_e_, where τ_e_ is the relaxation time that depicts the onset of entanglements among the polymer chains, until it is no longer visible in the experimental window. We can also observe ω_d_ shifting to lower frequencies with increasing concentration implying the increase in the density of entanglements as HA concentration is increasing. It can be seen that the system transitions from a frequency dependent to a relatively frequency independent behavior at high concentrations indicating a material dominated majorly by elasticity. The material reaches a viscoelastic solid-like state at the highest concentration of 18 wt %.

Upon further increase in the molecular weight to a high range of 750–1000 kDa (HA_H_), the terminal regime disappears from the experimental window (Figure 2C). At the lowest studied HA_H_ concentration of 0.1 wt %, *G*″ although entirely above *G*′ does not follow Rouse dynamics, but instead depicts a power-law behavior with an exponent of 0.57 (in good solvent), indicative of a dilute regime (Zimm dynamics).^43,44^

### Prediction of Tack from Linear Rheological Properties

The performance of adhesives, particularly their bonding strength, has been extensively studied, revealing a strong correlation with their bulk viscoelastic properties.^43,45–47^ Empirical and theoretical relationships have been established that link linear rheological properties to key adhesive characteristics. Chang developed so-called viscoelastic windows based on the *G*′ and *G*″ values using 10^–2^ and 10^2^ rad/s as the frequency windows.^48^ These viscoelastic windows were proposed to fall into 4 quadrants: Q1 non-PSAs (top-left; high *G*′ and low *G*″); Q2 high shear PSAs (top-right; high *G*′ and high *G*″); Q3 removable medical PSAs (bottom-left; low *G*′ and low *G*″); and Q4 quick stick PSAs (bottom-right; low *G*′ and high *G*″). As can be seen in Figure 3A, PSAs prepared with HA_L_ are entirely present in Q3 characterized by low *G*′ and low *G*″. This indicates their potential to be used as medical adhesives since they can be removed easily without damage to the substrates due to their low peel strengths. However, it is also important that the moduli should not be too low to ensure creep-resistance. Upon increasing the concentration of HA_L_, the viscoelastic windows move toward Q2 in a diagonal fashion indicating an increase in their peel strengths. Even at the highest studied concentration of 6 wt %, the viscoelastic windows are well within Q3. When the molecular weight is increased to HA_M,_ we observe that the windows get smaller and more square-like as can be seen in Figure 3B. Upon increasing the concentration past 6 wt %, it can be seen that the windows transform slowly to the Q1 region indicative of non-PSA like behavior. This makes the material ineffective as PSAs owing to their high elasticity combined with low loss modulus. This undesirable trend indicates that increasing the polymer concentration further is no longer beneficial since it leads to a loss of dissipative abilities (*G*″) combined with an increase in elasticity (*G*′). Interestingly, irrespective of the HA molecular weight, an increase in concentration led to the reduction in the size of the viscoelastic windows indicative of *G*′ and *G*″ having similar values due to their frequency independent behavior at high concentrations.

**Figure 3:**
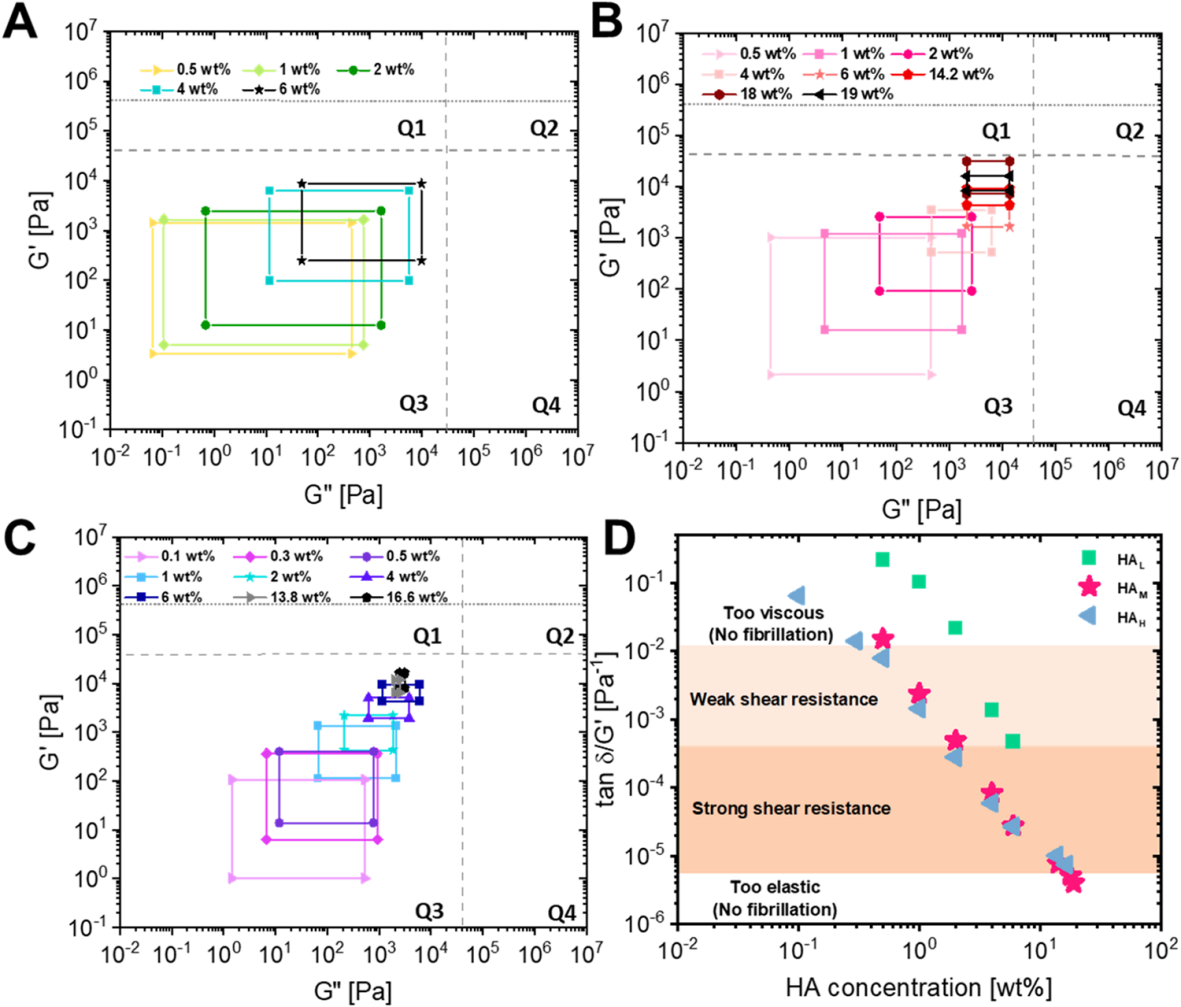
Chang′s viscoelastic windows predicting tack properties for HA in GFSH with molecular weights of (A) 30–50 kDa (HA_L_), (B) 200–400 kDa (HA_M_) and (C) 750–1000 kDa (HA_H_). Dashed gray lines represent the limits of the four quadrants and the dotted gray line represents the Dahlquist’s criterion. (D) tan δ­(ω)/*G*′(ω) vs. HA concentration at a debonding frequency of 3.98 rad/s to estimate the tendency for fibrillation (the formation of filaments during debonding), which contributes to energy dissipation.

To better predict the tack properties, several other criteria have been established. One such predominantly used criterion is the Dahlquist condition, as indicated by the dotted line in Figure 3A–C, which stipulates that the storage modulus of the adhesive should be within an upper limit of 10^5^ Pa at the bonding frequency.^49^ This is hypothesized to be essential to establish good contact with the substrate within the time provided for contact formation. In this study, all studied materials exhibited a storage modulus less than 10^5^ Pa, particularly also at the bonding frequency of 0.17 rad/s (corresponding to a contact time of 1 min), indicating that they remain sufficiently soft for effective contact formation. However, further increasing the HA concentration to increase *G*′ even more does not seem beneficial, as the loss modulus (*G*″) does not increase proportionally, which may limit energy dissipation and adhesive performance.

Another criterion was proposed by Deplace et al.^47^ The ratio tan δ­(ω)/*G*′(ω) (where tan δ = *G*″/*G*′ representing the dissipative potential of a material) was proposed as an approximation for this purpose with parameters that can be directly obtained from linear rheological measurements. This ratio combines the bulk effects contributing to elasticity with the resistance to crack propagation imparted by the viscous effects. In order to estimate the value of the debonding frequency ω, the following equation was used

$$\omega =\frac{2{\pi V}_{deb}}{h_{0}}$$

where *V*_deb_ is the retraction velocity of the probe (100 μm/s) and *h*_0_ is the initial sample thickness (150 μm).

The ratio tan δ­(ω)/*G*′(ω), was thus plotted at a debonding frequency of 3.98 rad/s which was the closest to the calculated value (4.19 rad/s) from the linear rheology data. This ratio serves as a predictive metric for energy dissipation during debonding in the probe tack test. Deplace proposed a limit below which energy dissipation does not take place with a viscoelastic PSA during debonding from a stainless-steel probe. It can be seen from Figure 3D, that most samples appear to fall above this limit with an exception to the highest concentrations of HA_M_ and HA_L_ lying right at the boundary. When materials have a tan δ­(ω)/*G*′(ω) ratio lower than this proposed limit, it is expected to be too elastic along with a very low dissipation ability resulting in quick crack propagation accompanied by low work of adhesion.^47^ An upper limit is difficult to be agreed upon since large values of tan δ­(ω)/*G*′(ω) indicates that the adhesive is dominated by liquid-like properties with very high dissipation. Although this would satisfy both the Dahlquist’s and Deplace criteria, it is important to remember that PSAs should be resistant to shear and debonding and hence, this also imposes an upper limit which is system dependent.

### Adhesive Properties

While linear rheology provides valuable insights, it only partially predicts adhesive performance since it does not entail nonlinear behavior, typically observed for adhesive applications. Predictions like the Deplace criteria were formulated for covalently cross-linked systems and not for such supramolecular systems. Hence, probe tack tests were employed to assess and compare the adhesion performance. Linear oscillatory measurements and nonlinear strain experiments have been utilized previously successfully for gaining information about various parameters such as the mode of debonding and work of adhesion.^43,50^

For most studied concentrations of HA_L_ in GFSH (<4 wt %) in Figure 4A, we observe very low forces required for debonding. The measured forces are mainly composed of capillarity and viscous forces arising from the liquid and viscous nature of these samples. This is in corroboration with the oscillatory measurements, where ω_d_ is absent for these materials, indicating the absence of any entanglements that can contribute to cohesiveness in the material. At 4 wt %, which is considered to be a semidilute unentangled state, we can see a larger area and a slight change in the shape of the stress vs. strain curve owing to the higher density of polymer chains at the border of forming entanglements. For a concentration of 6 wt %, there is a significant shoulder present reminiscent of the energy needed to overcome the entanglements in the material (as can be confirmed from Figure 2A at the approximated debonding frequency of 3.98 rad/s). This is also depicted in Figure 4D where the adhesion work remains relatively low and similar across the dilute regime (up to 2 wt %), beyond which there is a significant increase, highlighting the necessity of high polymer concentration for effective adhesion.

**Figure 4:**
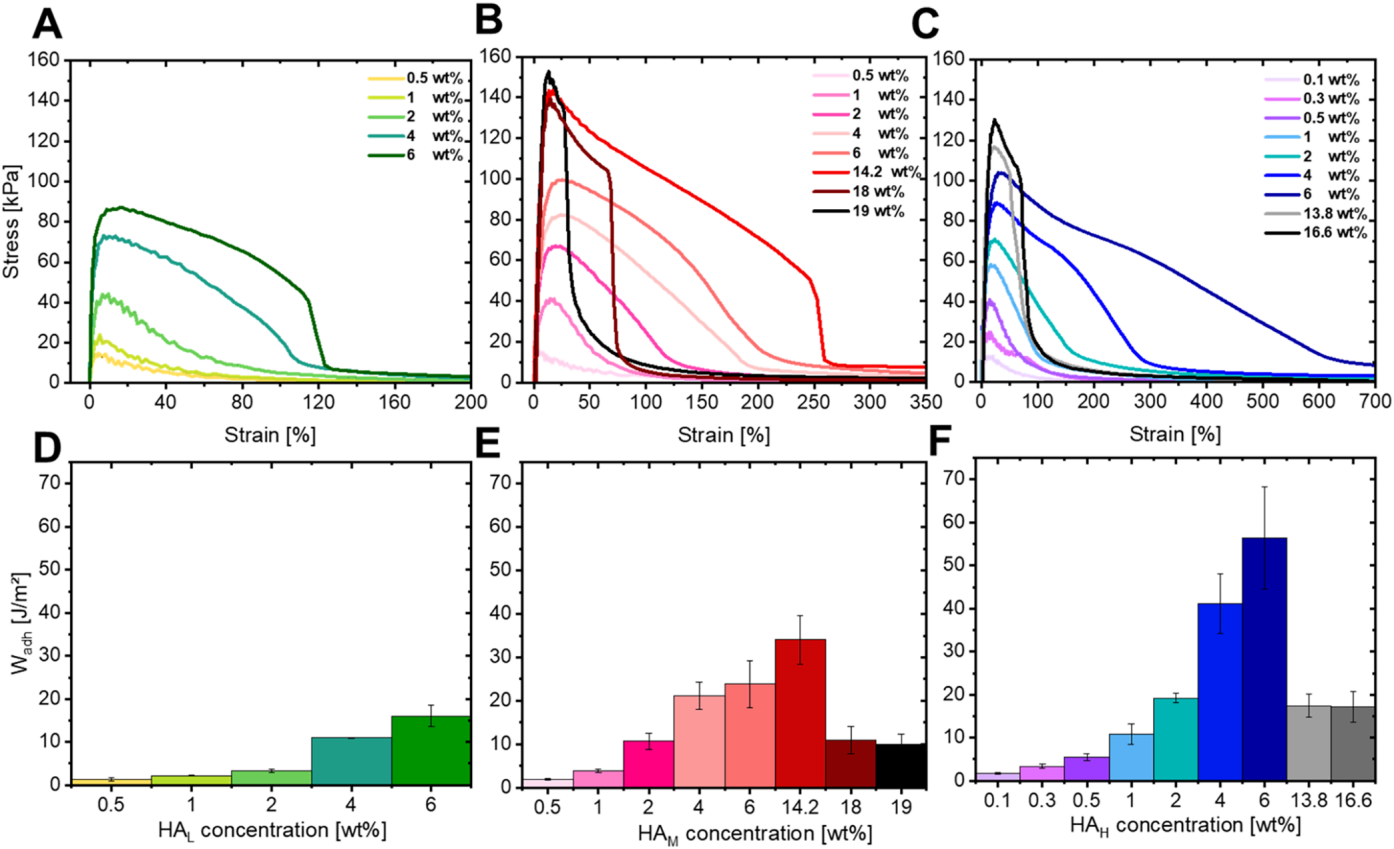
Adhesion measurements on HA in GFSH showing stress vs. strain during retraction for (A) 30–50 kDa (HA_L_), (B) 200–400 kDa (HA_M_) and (C) 750–1000 kDa (HA_H_) with corresponding work of adhesion shown for (D) 30–50 kDa (HA_L_), (E) 200–400 kDa (HA_M_) and (F) 750–1000 kDa (HA_H_).

An increase in the molecular weight to HA_M_ in GFSH interestingly displays much better adhesion performance as can be seen in Figure 4B. For the lowest concentration of 0.5 wt %, the stress vs strain curve closely resembles in shape to the lowest concentrations of HA_L_, indicating that all the energy is undesirably dissipated rather quickly through capillarity and viscous forces. However, from a concentration of 2 wt %, we see the emergence of a shoulder arising from the entanglements sustaining the applied stress, thereby increasing the cohesiveness of the material. This is also visualized in the increase of the nominal peak stress with increasing polymer concentration. Upon further concentrating the polymer network at 18 wt %, we observe a sudden large drop in the area under the stress vs strain curve which is also reflected in the drop in the work of adhesion values in Figure 4E. It can be understood that further increase in polymer concentration negatively influences the adhesive performance due to increased elasticity, which is in corroboration with the Chang’s viscoelastic windows in Figure 3B where the windows move toward Q1 due to loss in dissipative ability. This results in a notable cohesive nature of the material which is also indicated by the similarity in peak stress between 14.2, 18, and 19 wt % HA_M_. However, optimal performance is achieved through a balance between cohesiveness and adhesiveness of a viscoelastic material, i.e., these materials increase greatly in terms of their elastic network, but undergo a loss in their viscous component, affecting their overall adhesive performance undesirably.

At the highest molecular weight, we observe a rapid increase in the adhesion performance despite Chang’s viscoelastic windows predicting similar behavior to the medium molecular weight system in Figure 3. This highlights the need for nonlinear tack measurements to accurately predict adhesive behavior in contrast to purely using linear measurements. Nonetheless, we also see a pronounced increase in the area under the stress vs. strain curves with increasing HA_H_ concentration as shown in Figure 4C. When the system has reached a regime where the polymer is so densely packed that additional chains make the material more elastic, there is a sudden drop in the work of adhesion as shown in Figure 4F, similar to the HA_M_ case. However, the reduction occurs already at 13.8 wt % compared to 14.2 wt % HA_M_ still showing improved adhesiveness. This is expected owing to the length of the HA_H_ chains, implying that the limit at which the elasticity of the material becomes undesirable for adhesiveness occurs at a lower concentration than for shorter HA_M_ chains.

An interesting feature to be noted is the increase in the strain at break from Figure 4A–C. When we consider a specific concentration, for example 6 wt %, the strain at break for the HA_H_ chains is approximately 5 times that of HA_L_. This is a clear indication of the influence of longer chains on increasing stretchability before failure, provided that the polymer concentration does not exceed the limit causing adhesive failure. Within this limit, chain entanglements could possibly restrict the movement of individual chains and allow the material to stretch further before breaking, as they can better distribute stress throughout the polymer network.^51,52^ We note that strain hardening, a characteristic commonly observed in many PSAs, is absent in our formulations. This behavior suggests that the network lacks architectural features that sufficiently restrict chain mobility on the time scale of the pulling deformation rate. In other words, mechanisms such as entanglements or cross-links, whether covalent or supramolecular, with relaxation times longer than the time imposed by the strain rate, appear to be minimal or absent. Instead, the mechanical response seems to be governed primarily by physical interactions with short relaxation times, such as hydrogen bonds which do not significantly resist large deformations under our testing conditions.

Visualizing the debonding mechanism at the interface has been proven to help in gaining a systematic understanding on the balance between viscous and elastic effects of an adhesive.^53,54^ Figure 5 and Video S1 shows a comparison of the debonding mechanisms between HA_L_, HA_M_, and HA_H_ at a concentration of 6 wt %. In the first case with the shortest polymer chains, debonding is initiated by the formation of numerous cavities in the center of the adhesive-substrate interface.^55,56^ As retraction continues, these cavities expand. However, this expansion appears to be parallel to the surface at this stage. The cavities quickly coalesce and grow outward due to not having stiff enough boundaries resulting in a large void in the middle. This material eventually fails cohesively with material flowing along the boundary of this void due to its high viscous nature as can be seen by the bright border of the void at the end of retraction in Figure 5A and in the side view of the HA_L_ samples in Video S2.

**Figure 5:**
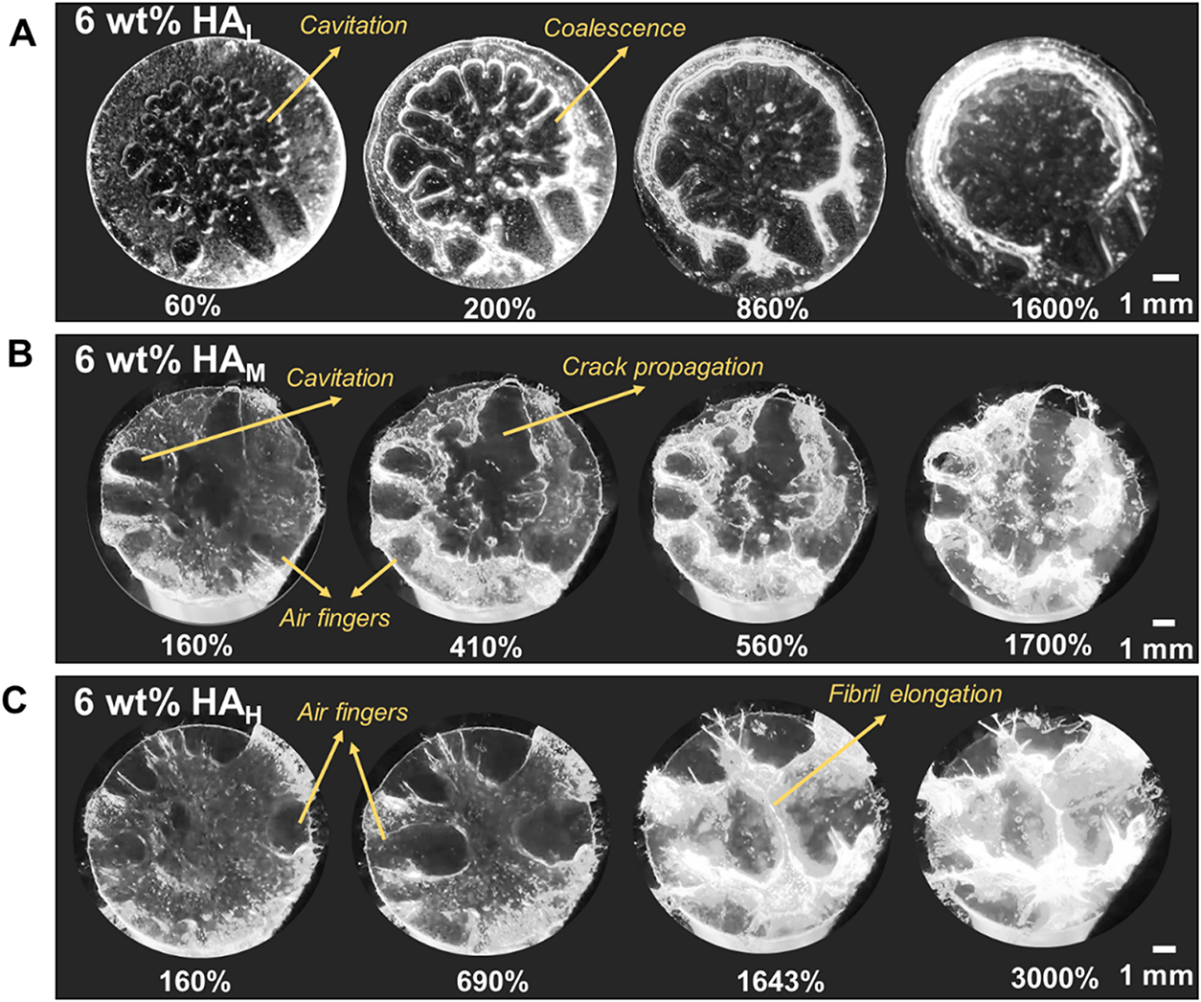
Bottom view during retraction at different strain percentages for HA in GFSH showing a transition from liquid-like to elastic nature while still exhibiting fibrillation across compositions (A) 6 wt % 30–50 kDa (HA_L_), (B) 6 wt % 200–400 kDa (HA_M_), and (C) 6 wt % 750–1000 kDa (HA_H_).

For the same polymer concentration, but with medium molecular weight chains, a drastically different debonding mechanism can be visualized in Figure 5B. Debonding initiates with cavitation observed at the interface as before. This is accompanied by the presence of fingering features which are typically instabilities that occur when a less viscous fluid (air in this case) injects or invades a more viscous fluid resulting in an unstable interface causing “air fingers”.^53,57^ We can also observe the coexistence of two debonding mechanisms; the fingering instabilities and crack propagation. Crack propagation involves the progressive failure of the adhesive interface, where the crack front advances through interfacial separation.^54^ Upon further extension, no significant change is observed in the bottom view indicating that bulk dissipation is taking place via the deformation of fibrils as can be confirmed from Video S1 and the side view of HA_M_ samples in Video S3.

At the highest studied molecular weight, debonding starts primarily due to the formation of large air fingers which appears to transition to a crack propagation mode of debonding as can be seen in Figure 5C. However, due to the increased stiffness of this material, the voids do not coalesce and instead, maintain their boundaries. Once they have expanded to their maximum limit without coalescing in the plane, the walls of the voids start elongating. This results in large bulk deformation perpendicular to the plane.^1,55^ This is a result of high resistance to crack propagation leading to energy dissipation via bulk deformation. The material eventually fails with most of the fibrils breaking at the interface rather than in the bulk.

These differences in behavior are further emphasized in Figure 6 and Video S1 by visualizing the retraction process of the 18 wt % HA_M_ and 13.8 wt % HA_H_; adhesives that were seen to perform poorly due to increased elasticity. In the former case, as shown in Figure 6A, we can observe a case of external crack propagation (from outside to inside) debonding the adhesive from the probe-adhesive interface in a clean fashion.^54^ It can be noted that there is no significant difference between the bottom view images throughout the retraction process indicating that the material is too elastic for the formation of air fingers and fibrils. In a similar fashion, Figure 6B shows a poor performing adhesive with HA_H_ chains, where we can also see a clear external crack propagating, but in the adhesive-base plate interface.

**Figure 6:**
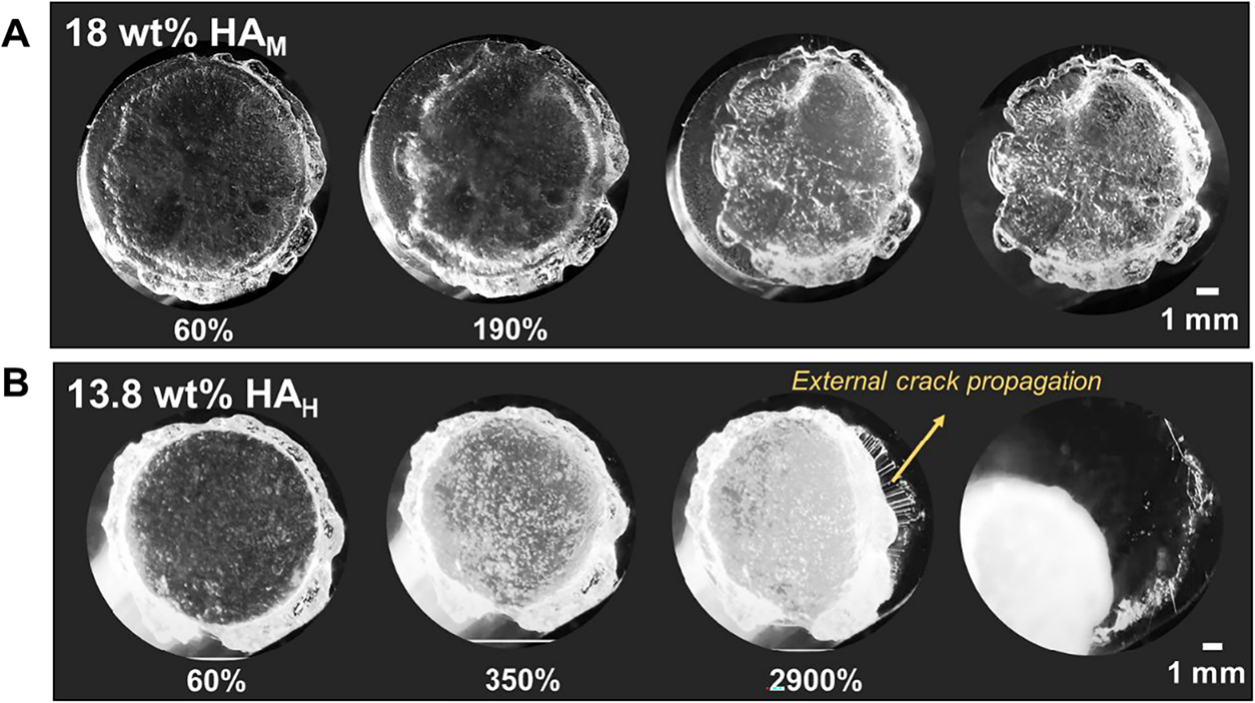
Bottom view during retraction at different strain percentages for HA in GFSH showing elastic materials across compositions (A) 18 wt % 200–400 kDa (HA_M_) and (B) 13.8 wt % 750–1000 kDa (HA_H_).

Based on the experimental observations of different debonding mechanisms, 2 regions were marked in the predictions of Figure 3D: a strong fibrillation region and a weak fibrillation region. Weak fibrils were characterized by soft 'flowing fibrils' that appeared to flow and coalesce together during retraction. The lower limit that characterizes the occurrence of fibrillation or interfacial crack propagation appears to be in good agreement with the Deplace limit (tan δ­(ω)/*G*′(ω) > 0.5 × 10^–5^ Pa^–1^). However, instead of having a rigid upper limit according to predictions, we observe a soft transition of the fibrillation region from strong fibrils to weak flowing fibrils before it reaches the upper limit of being dominated by purely viscous and capillary forces. It can however be expected that materials in the weak fibrillation region would not function as high shear PSAs since they lack sufficient creep resistance, a characteristic feature of conventional PSAs.

An interesting feature to be noted are the presence of very thin fibrils for the 13.8 wt % HA_H_ sample, as seen in Figure 6B and in the side view from Video S4. This could be contradictory to our assumption of an elastic material since crack propagation is the major debonding mechanism. It appears to be that the material owing to its highly viscous state, could have some inhomogeneities where viscous property dominates. However, it is important to note that all measurements are reproducible indicating that the material along with its inhomogeneities could exist in a kinetically trapped state giving rise to local regions where fibrillation occurs. Additionally, we recognize that geometrical confinement effects arising from varying film thickness near the adhesive-substrate edge may also induce local stresses and fibril formation. Nevertheless, these fibrils are very weak and thin hinting that crack propagation still remains as the primary debonding mechanism and although the material is still relatively viscoelastic, the elastic properties indeed dominate over its viscous nature. This phenomenon is also observed in the case of 18 wt % HA_M_. Although these are not visible in Figure 6A, it can be visualized in the side view in Video S3. All bottom view videos can be compared in Video S1.

### Analysis of Tack Properties

A striking feature in the work of adhesion data is the similarity in the trend for all three molecular weights. Disregarding the highest concentrations, it is evident that in the range where increasing polymer content still enhances performance, the adhesion work exhibits similar slopes irrespective of molecular weight as shown in Figure 7A. This implies that the physical interactions that contribute to adhesiveness at a molecular level scale linearly with the increasing number of monomer units. In order to identify the origin of these interactions, we can take a closer look at the retraction curves by extracting the peak stress as a function of the HA concentration in Figure 7B, we observe that each molecular weight depicts two regions with different slopes. In the first region, peak stress steeply increases with increasing HA concentration. However, peak stress for all molecular weights converge to similar values from 4 wt % for HA_L_, 2 wt % for HA_M_, and 1 wt % for HA_H_ (dashed arrows in Figure 7B), leading to the second slope region.

**Figure 7:**
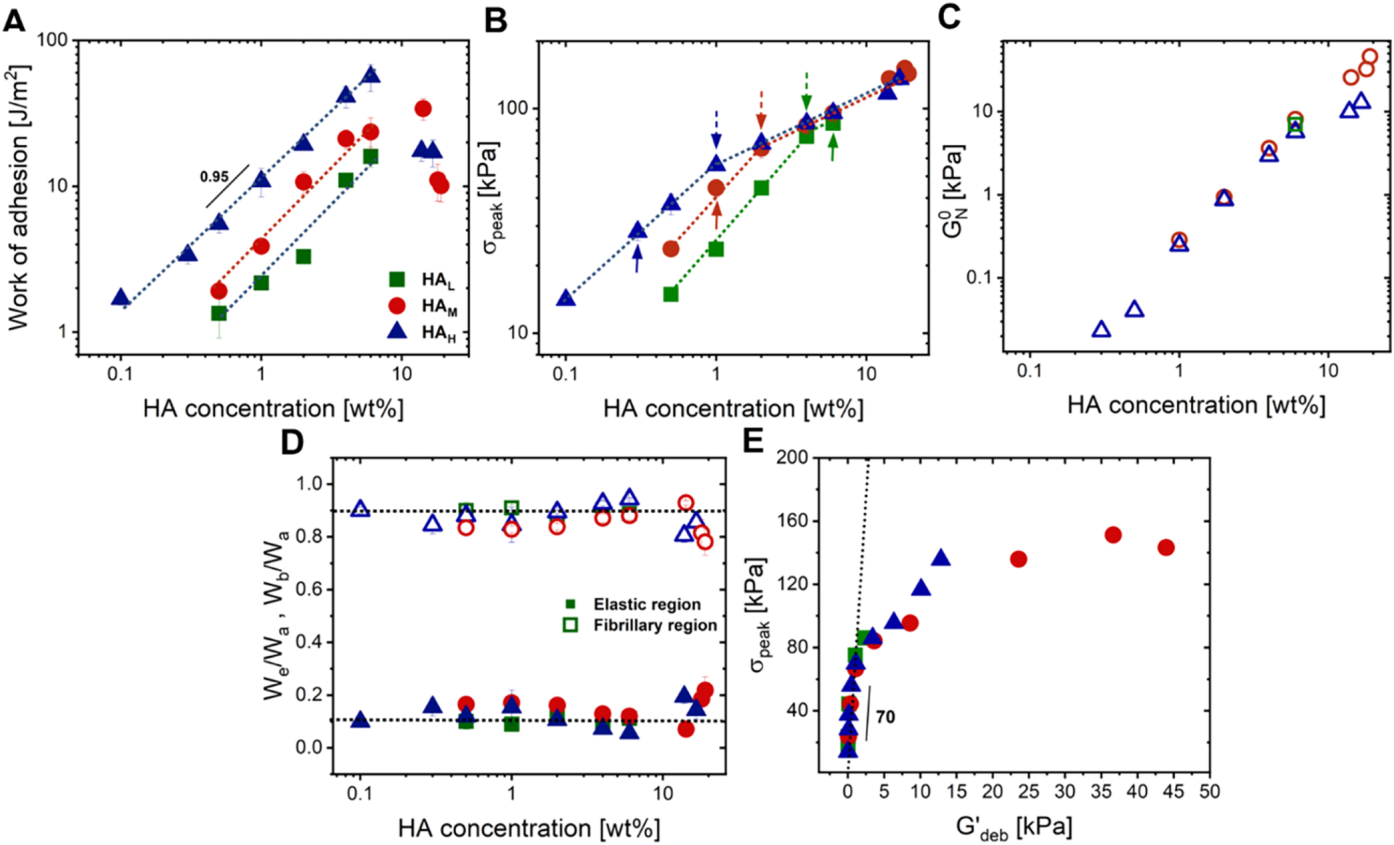
(A) Work of adhesion, (B) peak stress, (C) plateau modulus, and (D) ratio of peak stress to plateau modulus, as a function of increasing HA concentration in GFSH. Solid arrows and dashed arrows in (B) indicate the onset of entanglements and onset of convergences for the corresponding molecular weight, respectively. (D) Fraction of elastic (*W*_E_) and fibrillary (*W*_F_) region contributing to the total work of adhesion (*W*_A_). (E) Peak stress from retraction as a function of the elastic modulus at the debonding frequency. Dotted lines are meant to act as a guide to the reader.

The analysis of the plateau modulus evolution,*G*_N_^0^ (value of the storage modulus corresponding to the lowest value of tan δ­(ω)), as a function of polymer concentration in Figure 7C can help further in understanding the reason for this convergence. It is well-known that the plateau modulus becomes independent of the molecular weight in an entangled regime.^40^ Once the polymer concentration is high enough to induce entanglements, the elastic behavior in the material is dictated by the density of these entanglements rather than the length of the polymer chains themselves. As long as the molecular weight of the chain is above the critical entanglement molecular weight *M*_e_, the plateau modulus being a bulk property remains constant at a particular concentration independent of the chain length, reflecting no change in the overall network. Upon comparison with the trend in peak stress in Figure 7B, it can be understood that the dominance of entanglements (indicated by solid arrows) can be linked to the onset of convergence (indicated by dashed arrows). If we compare a particular concentration, 1 wt %, between all molecular weights, HA_L_ depicts a terminal regime, HA_M_ depicts an onset of entanglements, while a clear entanglement plateau can be observed for HA_H_ (as can be seen in Figure 2). This translates to peak stresses increasing respectively at these concentrations.

However, a more subtle observation from Figure 7B is that the point of convergence in peak stress (dashed arrows) does not align perfectly with the onset of the entanglement plateau (solid arrows) observed from SAOS. This mismatch may stem from the presence of weak supramolecular associations, potentially between the NaDES and HA, that transiently link the HA chains and elevate the apparent elasticity in the linear regime. These associations may contribute to the early appearance of the rubbery plateau in linear rheology, particularly for HA_M_ and HA_H_. Yet, under the large deformations probed by tack tests, these weak interactions likely dissociate, meaning that only true physical entanglements contribute to the observed peak stress. This interpretation is supported by the observation that peak stress increases sharply in the first region of Figure 7A when the material transitions from a primarily viscous to a more elastic regime. However, once physical entanglements are well established (second slope region in Figure 7B), further increases in elasticity have a smaller effect on peak stress. In essence, peak stress is strongly influenced by the elastic component of the material. The absence of a visible mismatch between slope change and plateau onset for HA_L_ may relate to its molecular weight being too low relative to *M*_e_, making entanglement contributions inherently limited in this system.

To gain further insight into the differences in debonding mechanisms between materials, the total work of adhesion (*W*_A_) from a typical stress vs. strain curve from probe tack tests was divided into an elastic region (*W*_E_ = *h*_0_∫_0_^ε_p_^σdε, where ε_p_ is the strain corresponding to σ_peak_) and fibrillary region (*W*_F_ = *h*_0_∫_ε_p__^∞^σdε).^58^ It is interesting to note from Figure 7D, that despite having such a broad range of HA molecular weights and concentration, the system appears to be entirely dominated by bulk deformation arising as a result of the fibrillary region. A significant change from the zero slope is observed at concentrations higher than 10 wt %. The fraction of fibrillation appears to reduce in this regime with a corresponding increase in the elastic region leading to interfacial crack propagation. This is in agreement with previous findings suggesting that at these concentrations, there is indeed a gain in the stiffness but is accompanied by a loss in the viscous nature of the materials.

It becomes important at this point to delve into the unique characteristics of the noncovalent network in these adhesives. An interesting finding in their mechanical response is the observed trend of peak stress vs *G*′_deb_ at high HA concentrations as shown in Figure 7E.^5^ It can be reasonably inferred that, for a specific probe surface and adhesive, the peak stress observed during retraction, is directly related to the cavitation stress, as this peak represents the formation of cavities across the majority of the surface. Initially, we observe a steep, nearly linear increase in peak stress with *G*′_deb_, indicating that elasticity plays a dominant role in peak stress build-up. The slope of this relationship (∼70) is much higher than that reported for commercial acrylic PSAs (∼10), suggesting that in our system, even small increases in linear elasticity lead to large gains in peak stress.^56^ However, this linear relationship does not hold at higher HA concentrations as the measured peak stress begins to deviate quickly from the predicted peak stress. This suggests that the system’s inherent softness, due to its high water content, reliance on entanglements, and weak supramolecular interactions, leads to early failure mechanisms such as interfacial cracking before peak cavitation stresses are reached.^56^ This highlights the difference between stiffness arising from the modulus and elasticity contributing to the peak stress. A material that has a high stiffness may not necessarily contribute to a high peak stress. It is necessary to note that while all the above approaches can act as very powerful tools for predicting adhesive properties for most PSAs, it is imperative to support them with nonlinear tack measurements since such approaches and predictions are not universal, especially in the case of soft adhesives.^43^

## Conclusions

In this work, a bioinspired PSA composed of natural sugars was developed and characterized. To the best of our knowledge, this is the first demonstration of a NaDES-based PSA, highlighting its potential as a sustainable alternative to conventional pressure-sensitive adhesives. These soft adhesives derive their properties primarily from hydrogen bonds and physical cross-links arising from polymer chain entanglements. We have studied the influence of HA imparting an elastic network within a viscous sugar-based DES giving rise to a tunable range of mechanical properties purely through changes in molecular weight and concentration of HA.

In addition, several concepts to predict adhesive properties from small amplitude oscillatory rheology that have been established for conventional PSAs were examined. We proposed the applicability of these frameworks to noncovalent adhesives and observed that longer HA chains and higher concentrations improve adhesive performance up to a certain limit. Debonding mechanisms at play in these regions include cavitation and fingering instabilities. Beyond this threshold, interfacial crack propagation becomes dominant, leading to a decline in adhesion. Although Chang’s viscoelastic windows offer a useful framework for qualitatively predicting adhesive performance, they categorize debonding processes into adhesive or cohesive failure without accounting for mixed modes of debonding. In our study, despite materials residing within the same viscoelastic quadrant, we observed significant variations in debonding behavior when visualized from the bottom and the side. In that regard, bulk dissipative abilities of different compositions could be divided into weak and strong fibrillation regions with the Deplace criteria. Although all materials exhibited stiffness below Dahlquist’s criterion, indicating their suitability as PSAs, a loss in dissipative properties ultimately rendered the highest polymer concentrations as poor adhesives. While these concepts showed strong correlation with our experimental results, nonlinear tack measurements remain essential to complement these predictions and provide a more comprehensive understanding of the debonding mechanisms in these systems, as evidenced in other studies.

While the bioinspired adhesives presented here show strong potential as sustainable PSAs, it is important to acknowledge current limitations that must be addressed for broader applicability. Due to their aqueous nature and reliance on physical cross-linking, these materials are inherently sensitive to environmental conditions such as changes in relative humidity. For long-term applications, especially those requiring creep resistance under stress, future work will need to explore stabilization strategies such as the addition of covalent cross-links to enable strain hardening. Nonetheless, the tunability and biocompatibility of this system make it promising for transient applications, where short-term adhesion and material safety are prioritized over durability.

Overall, the bioinspired adhesive developed in this study, being derived from nontoxic, renewable resources, presents a significant step forward in the design of environmentally friendly alternatives, especially for biomedical applications.

## Supplementary Material









## Notes

Bottom view of HA_L_, HA_M_, and HA_H_ during probe tack testing (Video S1) (MP4)
Side view of HA_L_ (30–50 kDa HA) over a concentration range of 0.5–6 wt % (Video S2) (MP4)
Side view of HA_M_ (200–400 kDa HA) over a concentration range of 0.5–19 wt % (Video S3) (MP4)
Side view of HA_H_ (750–1000 kDa HA) over a concentration range of 0.1–16.6 wt % (Video S4) (MP4)
